# Transcriptome Analysis of Key Genes Involved in Color Variation between Blue and White Flowers of *Iris bulleyana*

**DOI:** 10.1155/2023/7407772

**Published:** 2023-01-18

**Authors:** Lulin Ma, Yiping Zhang, Guangfen Cui, Qing Duan, Wenjie Jia, Feng Xu, Wenwen Du, Xiangning Wang, Xiang Li, Fadi Chen, Jihua Wang

**Affiliations:** ^1^Flower Research Institute of Yunnan Academy of Agricultural Sciences, National Engineering Research Center For Ornamental Horticulture, Key Lab of Yunnan Flower Breeding, Kunming 650205, China; ^2^College Horticulture of Nanjing Agricultural University, Nanjing 210095, China

## Abstract

*Iris bulleyana* Dykes (Southwest iris) is an extensively distributed Iridaceae species with blue or white flowers. Hereby, we performed a systematic study, employing metabolomics and transcriptomics to uncover the subtle color differentiation from blue to white in Southwest iris. Fresh flower buds from both cultivars were subjected to flavonoid/anthocyanin and carotenoid-targeted metabolomics along with transcriptomic sequencing. Among 297 flavonoids, 24 anthocyanins were identified, and 13 showed a strong down-accumulation pattern in the white flowers compared to the blue flowers. Significant downregulation of *3GT* and *5GT* genes involved in the glycosylation of anthocyanins was predicted to hinder the accumulation of anthocyanins, resulting in white coloration. Besides, no significant altered accumulation of carotenoids and expression of their biosynthetic genes was observed between the two cultivars. Our study systematically addressed the color differentiation in *I. bulleyana* flowers, which can aid future breeding programs.

## 1. Introduction

Southwest iris (*Iris bulleyana* Dykes) is a perennial plant of the genus Iris, widely distributed in southwestern regions of China, viz., Sichuan, Yunnan, and Tibet [1]. Iris (Iridaceae) genus, with over 300 species originating from Northern Hemisphere, is famous for its broad-spectrum palette of flower colors and patterns [2]. The name “iris” is derived from a Greek word with the meaning “rainbow.” There are about 60 species of iris plants in China. Most varieties are flower color variants, such as white-flowered: *I. tectorum* f. alba Makino [3], *I. sanguinea* Donn ex Horn. f. alba Makino [4], and *I. japonica* Thunb. f. pallescens PL Chiu et Y. T; dark-colored: *I. haynei* Baker, *I. petrana* Dinsmore, and *I. bostrensis* Mouterde; violet-colored: *I. ruthenica* Ker-Gawl. f. leucantha YT Zhao [5], *I. potaninii* Maxim. var. ionantha YT Zhao [6], and *I. lortetii* W. Barbey; blue-colored: *I. latistyla* YT Zhao f. albiflora J. Luo [7] and *I. lactea* Pall. var. chrysantha Zhao [8]; and yellow-colored: *I. halophila* var. sogdiana [9]. The subtle color variants of the Iris genus, ranging from dark purple, through blue, pink, and violet, to yellow and white flowers, have been the focus of scientists for many decades [10, 11].

Flowers tend to show colossal color variation within and between species [12–14]. Based on published reports, the identified pigments responsible for color variation in flowers can be categorized as carotenoids, flavonoids, and betalains pertaining to their synthesis, structures, and subcellular localization [15]. The synthesis of each pigment involved the interplay of multiple underlying genes [16]. Flavonoids from the phenylpropanoid class are secondary metabolites with a broad-spectrum color range, from pale-yellow to blue [16–18]. Particularly, anthocyanins, a subclass of flavonoids with a wide distribution in seed plants, have a major role in governing pigmentation in many flowers [16, 19–23]. Carotenoids are considered a vital component of the photosystem, and their subsequent expression confers yellow to red color in fruits and flowers [21, 24–26]. The coexistence of flavonoids/anthocyanins and carotenoids, resulting in rich coloration, has been described in many studies [27–31]. Flavonoids/anthocyanins and carotenoids are often present in the same organs, and their combination increases color variety. The synthesis pathways of these two types of pigments are well characterized [23, 26, 27, 32–35] and have been attributed to many plant species, i.e., *Arabidopsis thaliana* [36], *Rosa rugosa* [23], *Dianthus caryophyllus* [37], and *Dracocephalum moldavica* [38]. Betalains, water-soluble metabolites, yellow-to-red nitrogen-containing compounds are derived from tyrosine. However, the exclusiveness of the coexistence of betalains with flavonoids/anthocyanin in Caryophyllales (Caryophyllaceae) and Molluginaceae (Molluginaceae) has raised major taxonomic debate [16].

Iris plants are generally dominated by two types of pigments: flavonoids/anthocyanins and carotenoids. Blue-purple colors are mainly attributed to anthocyanin pigments, while orange, yellow, and pink colors are attributed to carotenoid synthesis. Various studies have identified multiple genes involved in the flavonoids/anthocyanins biological pathways for which alteration in gene expression induces color mutation. These genes include *CHS* (*chalcone synthase*) in parsley [39], petunia [40], tobacco [41], and safflower [42]; *CHI* (*chalcone isomerase*) in petunia [43], tobacco [44], and carnation [45]; *F3H* (*flavanone-3-hydroxylase*) in carnation [46], cineraria [47], saussurea [48], and peony [49]; *DFR- dihydroflavonol 4-reductase* in lily [50], gentian [51], peony [49], and saussurea [52]; *ANS* (*anthocyanidin synthase*) in gerbera [53] and peony [49]; *glycosyltransferase* (*GT*) in *Veronica persica* [54] and *Bellis perennis* [55]. Besides, some known transcription factors have also been reported to play a regulatory role in pigmentation, i.e., MYB, bHLH, and WD40 [56–58]. However, *Iris bulleyana* Dykes has not been characterized for its color formation. Due to its wide distribution in southwestern China and as a model species for studying the color formation, insight into the mechanisms underlying pigmentation will facilitate understanding the color formation and further breeding of colorful cultivars. Hereby, we have profiled the transcriptome and metabolome of Southwest iris (*I. bulleyana* Dykes) and its white variant (*I. bulleyana* Dykes f. alba YT Zhao) to pinpoint the genetic mechanism underlying flower color variation. Our study discussed the differential expression of key genes in carotenoids and anthocyanin biosynthesis pathways for their potential involvement in color formation in iris.

## 2. Results

**Figure 1:**
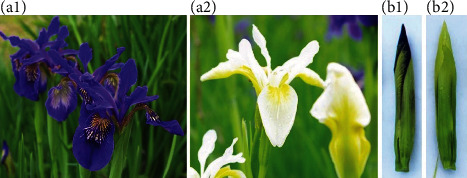
A1 and A2 are the blooming flowers of the Southwest iris and white-flowered iris, respectively; B1 and B2 are the flower buds of the Southwest iris (LHWY) and white-flowered iris (BHWY), respectively.

Southwest iris (*I. bulleyana* Dykes) generally has blue petals; however, another variant with white petals (*I. bulleyana* Dykes f. alba YT Zhao) is also present (Figure 1). To understand the genetic variation underlying this variation, we performed transcriptomic and targeted metabolomics following sample collection from Southwest iris and its white variant.

### 2.1. The Differential Landscape of Metabolites between Blue- and White-Colored Southwest Iris

Randomly selected fresh flower buds of Southwest iris and its white variant were collected and subjected to targeted metabolomics, revealing the differential landscape of metabolites, specifically anthocyanin and carotenoids.

Flavonoid profiling identified 297 metabolites with 69 differentially accumulated (25 downregulated and 44 upregulated in white flower samples compared to blue flowers) flavonoids between both flowers (Additional Files 1 and 2). A total of 24 anthocyanins were identified in the two groups of samples, and only 13 showed differential expression patterns between both flower types, and all 13 were downregulated in white flowers compared to blue flowers (Table 1). These anthocyanins included cyanidin 3-O-glucosyl-malonylglucoside, delphinidin O-malonyl-malonylhexoside, peonidin, cyanidin O-syringic acid, cyanidin 3-O-glucoside (kuromanin), delphinidin 3-O-glucoside (mirtillin), malvidin 3,5-diglucoside (malvin), delphinidin 3-O-rutinoside (tulipanin), pelargonidin 3-O-beta-D-glucoside (callistephin chloride), cyanidin 3-O-galactoside, peonidin 3, 5-diglucoside chloride, petunidin 3, 5-diglucoside, and peonidin 3-sophoroside-5-glucoside. Some of these anthocyanins were further validated using the LC-MS/MS standard-based quantification (Table 2). White variant showed less abundance of these three metabolites; particularly, delphinindin chloride and myrtillin (delphinidin 3-glucoside (Dp 3G)) chloride were not detected in white flowers. These results emphasized that either downregulation or blockage of anthocyanins in the white variant of Southwest iris is likely to be the major reason for differentiation from blue to white color.

Furthermore, carotenoid metabolites were also investigated in both groups. Eleven carotenoids were identified using targeted metabolomics (Additional Files 3, 4, and 5). There was no significant differential accumulation of carotenoids in blue and white flowers. However, *α*-carotene depicted higher accumulation in blue flowers compared to white, while zeaxanthin and xanthophyll (lutein) both up accumulated in white flowers. The changes in accumulation patterns of these three carotenoids were statistically nonsignificant, suggesting a neglected role of carotenoids in color differentiation from blue to white flowers. The accumulation pattern of carotenoids in purple and white flowers explained the conserved yellow stripes on both flowers.

### 2.2. Differential Landscape of Expressed Genes between Blue- and White-Colored Southwest Iris

**Figure 2:**
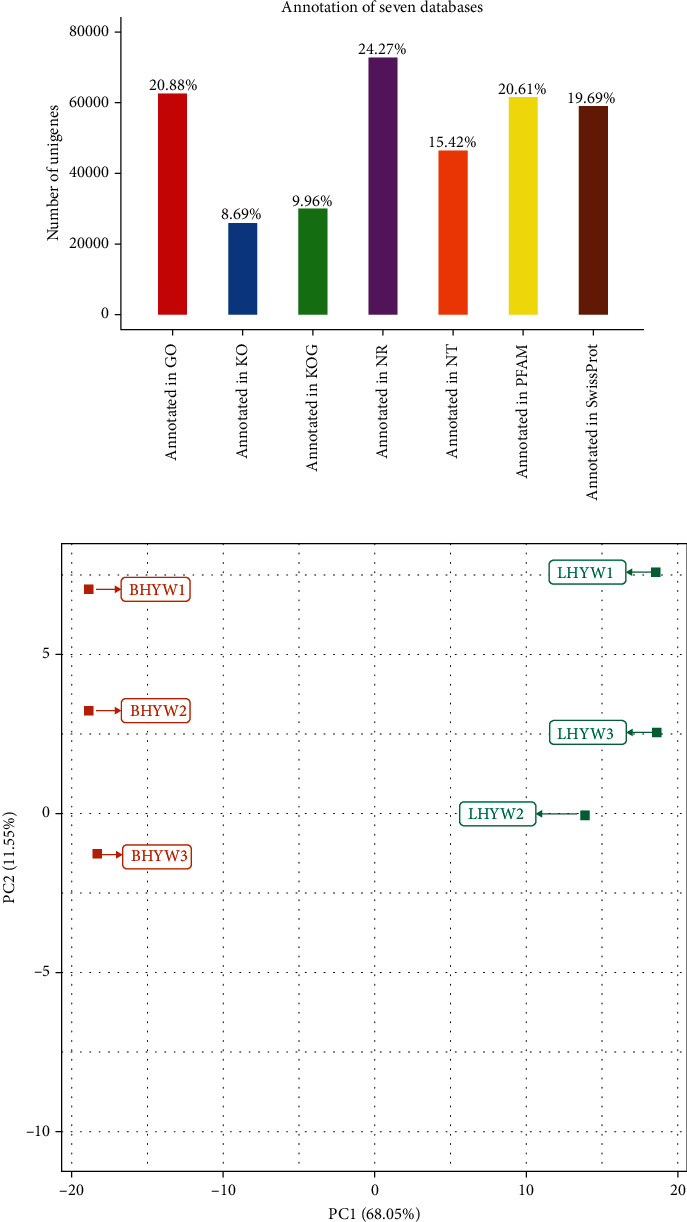
Transcriptomic analysis of differentially expressed genes (DEGs) between Southwest iris (LHWY) and its white variant (BHWY). (a) Bar plots representing the number of unigenes identified and annotated through multiple platforms, viz., NT, NR, KOG, GO, and PFAM. (b) PCA based on the FPKM values in LHWY and BHWY samples.

In order to analyze the metabolism of anthocyanins in different colors, two libraries were constructed with blue and white perianths during the full bloom period for high-throughput sequencing. The clean data of each sample reached 8.91 Gb, and the Q30 base percentage was higher than 91.76%. The GC contents of the white and blue flowers were 46.32 and 46.49%, respectively (Additional File 6). Through the above sequencing quality control, high-quality clean data were obtained and used for downstream analysis. Subsequently, 370,387 transcripts and 299,827 unigenes were recombined and annotated against seven databases, viz., NT, NR, KOG, GO, and PFAM (Figure 2(a)). Principal component analysis (PCA) differentiated both color variants into two groups, and biological replicates were closely grouped (Figure 2(b)). PCA results suggested high reliability of transcriptome data for further analysis.

### 2.3. Differential Expression between Blue and White Flowers of Southwest Iris

Based on differential expression analysis in Southwest iris and its white variant, a total of 422 differentially expressed genes (DEGs) were identified, with 242 upregulated and 180 downregulated genes in the blue flowers compared to the white flowers (Additional File 7). The identified DEGs depicted significant enrichment in phenylpropanoid biosynthesis, flavonoid biosynthesis, pyrimidine biosynthesis, and photosynthesis. Accumulating KEGG annotation and DEGs, we identified 21 genes associated with flavonoid/anthocyanin biosynthesis and carotenoid biosynthesis (Table 3). Three genes, viz., *c174379_g1* (3GT (anthocyanin 3-O-glucosyltransferase)), *c178689_g1* (CHS (chalcone synthase)), and *c134319_g1* (5GT (anthocyanin 5-O-glucosyltransferase)), showed downregulation expression pattern in the white variant as compared to the blue flowers. While other genes *c165047_g2* (*CHS2* (chalcone synthase 2)), *c151362_g1* (*CHI* (chalcone-flavonone isomerase)), *c173776_g1* (*FNS* (flavone synthase)), *c145508_g1* (*F3H* (flavanone 3-hydroxylase)), *c144091_g2* (*F3*′*H* (flavonoid 3 ′-hydroxylase)), *c144091_g1* (*F3*′*5*′*H* (flavonoid 3′,5′-hydroxylase)), *c144091_g1* (*FLS* (flavonol synthase)) *c151171_g1* (*DFR* (dihydroflavonol-4-reductase)), *c117196_g1* (*ANS* (anthocyanin synthase)), *c161528_g2* (*5,3GT* (glucosyltransferase)), *c167438_g1* (*3AT* (3-Amino-1,2,4-triazole)), *c105331_g2* (*URT*-UDP-rhamnose: anthocyanidin 3-*O*-glucoside rhamnosyltransferase), *c160759_g1* (*PSY2* (phytoene synthase)), *c172816_g1* (*PDS* (phytoene desaturase)), *c168460_g1* (*ZDS* (*Z*-carotene desaturase)), *c168442_g1* (*LCYB* (lycopene *β*-cyclase)), *c144636_g1* (*ZEP* (zeaxanthin epoxidase)), and *c143882_g1* (*VDE* (violaxanthin deepoxidase)) did not show a significant differential expression between the two variants. These results further confirm that the carotenoid biosynthesis pathway has no important effect on the white/blue flower coloration. Besides, the downstream product of *CHS* (chalcone substance) has little change between the two flower types, indicating that the flower color variation observed in the Southwest iris is mainly affected by the sharp downregulation of *3GT* and *5GT* genes.

Based on previously published reports suggesting the involvement of MYB and bHLH transcription factors as a key regulators in plant pigmentation [56–58], we identified 158 MYBs and 122 bHLHs. However, their expression was conserved between the Southwest iris and its white variant.

### 2.4. Proposed Mechanisms of Blue/White Color Formation in Southwest Iris

**Figure 3:**
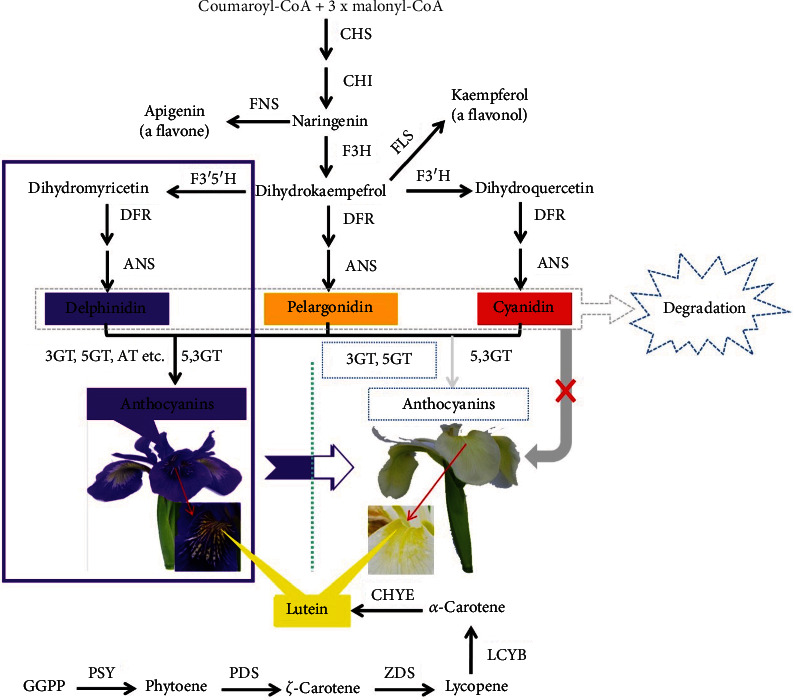
Schematic diagram of differential biosynthesis of pigmentation in Southwest iris where *3GT* and *5GT* downregulation shunted the anthocyanin pathways and resulted in white phenotype. Anthocyanin biosynthesis has been represented through a series of catalysts, including *CHS* (chalcone synthase), *CHI* (chalcone isomerase), *F3H* (flavanone-3-hydroxylase), *F3*′*H* (flavonoid 3′-hydroxylase), *F3*′*5*′*H* (flavonoid 3′,5′-hydroxylase), *DFR* (dihydroflavonol 4-reductase), *ANS* (anthocyanidin synthase), *3GT* (anthocyanin 3-O*-*glucosyltransferase), and *5GT* (anthocyanin 5-O-glucosyltransferase). No significant differential expression was observed for the above-mentioned genes except for *3GT* and *5GT*, with downregulated expression pattern in white flowers resulting in the down-accumulation of 13 anthocyanins. No significant change in the carotenoid pathway explains the conserved yellow stripes in the flowers of both genotypes (PSY2 (phytoene synthase), PDS (phytoene desaturase), ZDS (*Z*-carotene desaturase), LCYB (lycopene *β*-cyclase), and CHYE (carotenoid *ε*-hydroxylase)). The genes colored in green exhibited normal expression between the two genotypes, while the genes colored in red were downregulated in the white variant. The blue box is the main anthocyanin synthesis process in the blue iris flower.

In the two Southwest iris variants, we identified 13 anthocyanins differentially accumulated. The initial anthocyanins are very unstable and can easily degrade [59, 60]; therefore, they need to be glycosylated and transferred into vacuoles for pigmentation. The *3GT* and *5GT* genes play this function [61], and because they were significantly downregulated in the white flower of Southwest iris, anthocyanin glucosides could hardly be produced, resulting in no blue coloration. In contrast, the high activity of *3GT* and *5GT* in the blue Southwest iris favored the formation and accumulation of anthocyanin glycosides, contributing to the blue color of the flowers (Figure 3). We did not observe any change in the carotenoid pathway, which explains the conserved yellow stripes in the flowers of both genotypes (Figure 3).

**Figure 4:**
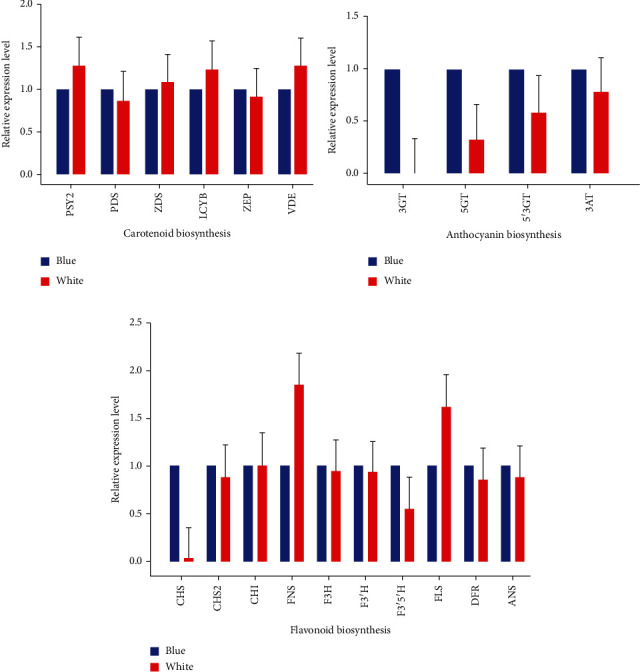
Relative expression profile of selected genes in Southwest iris (blue) and its white variant (white). (a) Carotenoid biosynthesis. (b) Anthocyanin biosynthesis. (c) Flavonoid biosynthesis.

To further confirm the expression of identified genes in the development of flower color, we performed qRT-PCR for three groups of selected genes related to flower color regulation, viz., flavonoid biosynthesis, anthocyanin biosynthesis, and carotenoid biosynthesis. The qRT-PCR results have been presented in Figure 4. Interestingly, the genes *3GT* and *5GT* showed significantly lower expression patterns in white flowers compared to blue flowers (Figure 4(b)), which further confirms our hypothesis that downregulation of *3GT* and *5GT* genes resulted in white coloration. Besides, genes related to carotenoid synthesis did not show significant differential expression in both flowers (Figure 4(a)), supporting our transcriptome results.

## 3. Discussion

Flower colors, with their eye appeal and aesthetic value, have been the focus of many biological studies [12–14], and genetic pathways for color development have been well characterized. Carotenoids, flavonoids, and betalains are primary metabolites characterized for their role in pigmentation in flower and fruit color. However, certain species-specific variations due to mutation, activities of regulatory genes, and multigene influence have also been reported [12–14, 35]. Therefore, this study was systematically designed utilizing metabolomics and transcriptomics to uncover flower color differentiation between Southwest iris (*I. bulleyana* Dykes) with blue flowers and its white variant (*I. bulleyana* Dykes f. alba YT Zhao).

Anthocyanins, a branch of flavonoids, have many biological functions in higher plants. Previously published literature suggested the essential role of anthocyanins in plant pigmentation. For instance, the red seed coat in peanuts has a strong association with anthocyanins [62]. A study by Qiu et al. demonstrated a significant increase in total anthocyanins in purple passion fruit compared to yellow [63]. White, yellow, blue, and pink *Primula vulgaris* [64] showed a gradual increase in total anthocyanin content as the color deepened. Moreover, anthocyanins play a critical role in plant defense responses against biotic and abiotic stress conditions [65, 66]. In iris, the presence/absence of anthocyanins is a critical factor for color development [19]. Flavonoid-targeted metabolomics identified 13 anthocyanins showing significant down-accumulation in white flowers compared to the blue flowers, which are predicted to favor the blue coloration. Cyanidin 3-O-glucosyl-malonylglucoside [67, 68], delphinidin O-malonyl-malonylhexoside [69], delphinidin 3-O-glucoside (mirtillin) [70–72], and delphinidin 3-O-rutinoside (tulipanin) have been previously reported for their active role in blue color pigmentation in perianths. Differential accumulation of anthocyanins pertaining to different flower colors and their corresponding shades has been reported in different iris species [73–76]. Further, anthocyanins, as biological/chemotaxonomic markers, have been used for the taxonomic classification ofspecies and cultivars [77, 78].

Dp3pCRG5G (delphinidin-3-pcoumaroylrutinoside-5-glucoside) is the most common anthocyanin in iris species and is generally responsible for blue-colored perianths is different iris species such as Dutch iris, Siberian iris, and *I. germanica* [19]. However, the precursor of DP3pCRG5G, delphinidin is very unstable [60], which requires further glycosylation for stabilization to the end product Dp3pCRG5G. Our transcriptome results suggest a downregulation of *3GT*-anthocyanidin 3-*O*-glucosyltransferase in the white flower [79]. The downregulation of the *3GT* gene is predicted to inhibit the synthesis of delphinidin 3-glucoside [80, 81]. Furthermore, a downregulation of another gene *5GT (*anthocyanidin 5-O-glucosyltransferase) was also observed in the white flower, which may result in reduced levels of delphinidin 3-rutinoside [82]. Florio et al., characterized acyltransferase, complemented by *5GT*, for differential accumulation of delphinidin-3-rutinoside and nasunin [82]. Contrary to our results, a study concerning gentian identified delphinidin 3,5,3′-O-triglucosideas a stable blue pigmentregulated by the coexpression of *3GT* and *5GT* [83]. Another study concerning rose petal coloration identified *5,3GT* as a contributor to petal coloration by catalyzing glycosylation at two different positions on anthocyanidin [84]. However, we observed a conserved expression of *5,3GT* in blue and white flowers. Interestingly, targeted metabolomics suggested a significantly higher accumulation of cyanidin 3-O-galactoside in blue flowers compared to white; however, we did not identify UDP-galactose: anthocyanidin 3-O-galactosyltransferase from the transcriptome data. UDP-galactose has been reported previously to influence the accumulation patterns of cyanidin 3-O-galactoside [85]. The reason for the differential accumulation of cyanidin 3-O-galactoside in the blue and white iris is unclear and requires further study to understand the accumulation pattern. Further insights into substrate recognition, utility, and structure-activity of *3GT* and *5GT* could provide significant results for pigmentation in the iris.

Moreover, we identified yellow stripes on both flowers, which were explained by similar accumulation patterns of carotenoids in purple and white flowers. Carotenoid biosynthesis has been well-documented in many plant species [34, 86, 87]. Yellow, orange, and red colors in plants are mainly attributed to carotenoid accumulation patterns [88]. A study concerning *Iris germanica* L. demonstrated the role of the *phytoene synthase gene* (*crtB*) in managing yellow color by increasing metabolite flux into carotenoid biosynthesis pathways [2]. However, in this study, there were no significant differences in accumulation patterns of carotenoids in purple and white flowers, explaining the conserved yellow stripes on both flowers. Moreover, the gene identified in carotenoid biosynthesis pathways depicted nonsignificant differences in purple and white flowers.

In contrast to our results, a recent report by Wang et al. [89] suggested a shunted anthocyanin pathway due to the absence of naringenin, a key compound in the pathways, as a major constraint in color differentiation from blue to white in *Iris laevigata* Fisch. However, in our study, naringenin chalcone was detected with a similar expression pattern of the corresponding *CHI* gene in both blue and white flowers, which highlights that various mechanisms are involved in the color variation in different Iris species.

Altogether, the down-accumulation of various anthocyanins, probably due to the strong downregulation of *3GT* and *5GT,* plays a major role in color differentiation between blue and white flowers in the Southwest iris. Further functional verification of these genes can provide a valid reference for the differential pigmentation pattern in the Southwest iris.

## 4. Materials and Methods

### 4.1. Plant Materials and Sample Collection

Wild Southwest iris, *Iris bulleyana* Dykes, and its white variant *I. bulleyana* Dykes f. alba YT Zhao were used in this study. *Iris bulleyana* Dykes grows naturally in the outskirts of Shangri-La county, Yunnan province, China. No permissions were necessary to collect such samples. The formal identification of the plant materials was undertaken by the corresponding author of this article. No voucher specimen of this material has been deposited in a publicly available herbarium. During its flowering stage, random samples from plants grown under a controlled environment were selected with the same conditions as the degree of development, size, and length. Flower samples were collected when half of the flower parts appeared from the bud (Figures 1(b1) and 1(b2)) after quickly removing the stalks and bracts at the base of the buds and placed in liquid nitrogen. Samples were stored at -80°C. The samples were collected with three biological replicates for each flower color, viz., blue (*Iris bulleyana* Dykes) and white (*I. bulleyana* Dykes f. alba YT Zhao). A total of six samples were used for transcriptome sequencing analysis, metabolome analysis, and qRT-PCR analysis.

### 4.2. Metabolic Profiling

The targeted metabolite landscape for flavonoids/anthocyanins and carotenoids was explored and analyzed according to the standard procedure detailed by Yuan et al. [90]. The flower samples collected from *Iris bulleyana* Dykes and *I. bulleyana* Dykes f. alba YT Zhao were grounded to powder and subjected to LC-MS analysis. UPLC-MS/MS analysis was performed by Metware (http://www.metware.cn). Prior to further data analysis, quality control (QC) analysis was performed. VIP (variable importance in projection) values were identified utilizing PLS-DA. The metabolites were considered differentially expressed when the VIP ≥ 1, and fold change ≥ 2 or fold change ≤ 0.5. To validate the anthocyanin metabolome, three selected anthocyanins were further tested using HPLC-MS/MS performed by Metware (http://www.metware.cn), and their corresponding concentrations were identified in both variants of Southwest iris.

### 4.3. RNA Extraction, Library Preparation, and Sequencing

Transcriptome sequencing was performed by constructing six libraries corresponding randomly collected bud samples, each with three replicates, of *Iris bulleyana* Dykes and *I. bulleyana* Dykes f. alba YT Zhao. After extraction of total RNAs with TRIzol reagent (Takara, China), contamination and RIN (RNA integrity number) were checked using 1% agarose gel and Agilent 2100 Bioanalyzer system (Agilent Technologies, CA, USA), respectively. Pair-end sequencing libraries were constructed using 3 *μ*g RNA for each sample. Further, libraries were generated using NEBNext® UltraTM RNA Library Prep Kit for Illumina® (NEB, USA) following manufacturer's instructions. Illumina HiSeq platform was utilized for RNA sequencing and was performed by the company Novogene (https://en.novogene.com/). The libraries were sequenced by paired-end sequencing on Illumina HiSeq.

Low-quality reads and short sequence reads (<50 bp) were removed using FastQC and Perl program. Clean reads were *de novo* assembled using Trinity v2.11.0 (http://trinityrnaseq.sourceforge.net). The transcriptome data of *Iris bulleyana* Dykes and *I. bulleyana* Dykes f. alba YT Zhao have been deposited to the national center for biotechnology information (NCBI) sequence read archive (SRA) under accession number PRJNA676187.

### 4.4. Differential Expression Analysis of Identified Genes

The read numbers mapped to each gene were counted using featureCounts v1.5.0-p3 [55]. Then, calculating the expected number of FPKM (fragments per kilobase of exon model per million reads mapped) of each gene based on the length of each gene and reads count mapped to the gene. DEGs between blue and white groups of colored samples were identified using the DESeq R package (v1.18.0) [91] and edgeR package (v 3.24.3). The threshold *p* value in multiple tests to judge the significance of gene expression difference was based on the false discovery rate (FDR) method. When FDR ≤ 0.05 and FPKM values showed at least a 2-fold difference among samples, the gene was considered a significant DEG. DEGs commonly detected by both packages were used in this study.

### 4.5. Validation of Gene Expression Using qRT-PCR

To verify the RNA-seq data, qRT-PCR was used following total RNA extraction from flower bud samples in three replicates, using the Tiangen RNAprep Pure Plant kit (Tiangen Biotech, Beijing, China), following the manufacturer's protocol. Twenty genes related to flavonoid/anthocyanin and carotenoid pathways of the transcriptome data were selected, and corresponding primers were designed for qRT-PCR using the Oligo-7 software (Additional File 8). The primers were synthesized by Sangon Biotech (Shanghai, China). Actin was used as an internal reference gene for qRT-PCR analysis of the target genes [92]. The cDNA was extracted from RNA and used as a template to make the reaction for qRT-PCR by using Takara qPCR kit SYBR Premix Ex TaqTM II (Tli RNaseH Plus). Three biological repeats were used for each qRT-PCR reaction.

### 4.6. KEGG Enrichment Analysis of DEGs

To test the statistical enrichment of Kyoto Encyclopedia of Genes and Genomes (KEGG) pathways, the GOseq R package was used. The KEGG pathways enriched with DEGs (FDR < 0.05) were detected using KOBAS 2.0 software [62] based on the method of overrepresentation analysis (ORA). The adjusted *p* value of significantly corroborated KEGG terms was less than 0.05.

## Figures and Tables

**Table 1:** Differentially accumulated anthocyanins in white and blue flowers. Values represent relative ion intensity.

No.	Anthocyanins	LHYW1	LHYW2	LHYW3	BHYW1	BHYW2	BHYW3	VIP	FC	LogFC
1	Cyanidin 3*-O*-glucosyl-malonylglucoside	6500	7470	5980	4210	9	9	1.39399	0.21193	-2.238
2	Delphinidin O-malonyl-malonylhexoside	36000	27200	31900	9	9	9	2.16576	0.000284	-11.782
3	Peonidin	135000	117000	111000	30700	44300	9	1.14918	0.206636	-2.274
4	Cyanidin O-syringic acid	1930000	2260000	1770000	9	9	9	2.65906	4.53*E* − 06	-17.751
5	Cyanidin 3-O-glucoside (kuromanin)	480000	509000	480000	9	9	9	2.50372	1.84*E* − 05	-15.731
6	Delphinidin 3-O-glucoside (mirtillin)	4480000	4770000	4070000	22600	23200	14700	1.76263	0.004542	-7.7824
7	Malvidin 3,5-diglucoside (malvin)	384000	438000	461000	9	9	9	2.48778	2.1*E* − 05	-15.536
8	Delphinidin 3-O-rutinoside (tulipanin)	74100000	81200000	85400000	645000	402000	433000	1.71279	0.006149	-7.345
9	Pelargonidin 3-O-beta-D-glucoside (callistephin chloride)	3570000	3530000	2790000	9	9	9	2.71313	2.73*E* − 06	-18.48
10	Cyanidin 3-O-galactoside	77300000	77100000	71400000	9	9	9	3.02707	1.2*E* − 07	-22.99
11	Peonidin 3, 5-diglucoside chloride	13900	21900	20300	9	9	9	2.09231	0.000481	-11.020
12	Petunidin 3, 5-diglucoside	1660000	1860000	1620000	126000	133000	104000	1.23394	0.070623	-3.823
13	Peonidin 3-sophoroside-5-glucoside	139000	131000	127000	9	9	9	2.34867	6.8*E* − 05	-13.843

^∗^BHWY represents the sample of the white variant of the Southwest iris (*I. bulleyana* Dykes f. alba YT Zhao), while LHWY represents the Southwest iris (blue).

**Table 2:** Determination of three anthocyanins in white and blue flowers by LC-MS/MS.

Sample	Delphinidin3-Orutinoside	Delphinidin chloride	Myrtillin chloride
BHWY-1	3.88	0	0
BHWY-2	4.38	0	0
BHWY-2	4.5	0	0
LHWY-1	372.1	436.58	5077.71
LHWY-2	2341.98	1446.2	17526
LHWY-3	1279.91	1031.9	12234

^∗^BHWY represents the sample of the white variant of the Southwest iris (*I. bulleyana* Dykes f. alba YT Zhao), while LHWY represents the Southwest iris (blue).

**Table 3:** Expressed genes in the flavonoid/anthocyanin and the carotenoid synthesis pathways based on transcriptome sequencing data of Southwest iris white and blue genotypes.

No.	Gene ID	Name	FPKM						Gene expression	Biosynthetic pathways
			LHYW1	LHYW2	LHYW3	BHYW1	BHYW2	BHYW3		
1	*c174379_g1*	*3GT*	1447.49	654.47	1108.74	0.84	1.13	1.63	Downregulated	Flavonoid/anthocyanin synthesis pathway
2	*c178689_g1*	*CHS*	233.67	458.13	297.36	1.61	2.21	1.79	Downregulated	Flavonoid/anthocyanin synthesis pathway
3	*c134319_g1*	*5GT*	420.46	93.77	204.55	31.35	46.71	85.09	Downregulated	Flavonoid/anthocyanin synthesis pathway
4	*c165047_g2*	*CHS2*	1879.22	1059.72	1352.54	413.99	630.32	1114.06	No difference	Flavonoid/anthocyanin synthesis pathway
5	*c151362_g1*	*CHI*	181.68	100.62	210.58	143.38	140.11	169.77	No difference	Flavonoid/anthocyanin synthesis pathway
6	*c173776_g1*	*FNS*	22.79	40.96	21.92	8.67	18.54	21.49	No difference	Flavonoid/anthocyanin synthesis pathway
7	*c145508_g1*	*F3H*	172.22	87.52	161.13	76.00	107.12	137.77	No difference	Flavonoid/anthocyanin synthesis pathway
8	*c144091_g2*	*F3*′*H*	17.29	13.65	16.73	4.34	10.64	17.24	No difference	Flavonoid/anthocyanin synthesis pathway
9	*c144091_g1*	*F3*′*5*′*H*	73.41	31.84	47.44	10.11	14.43	37.69	No difference	Flavonoid/anthocyanin synthesis pathway
10	*c172658_g1*	*FLS*	4.01	19.89	7.5	2.44	6.95	7.05	No difference	Flavonoid/anthocyanin synthesis pathway
11	*c151171_g1*	*DFR*	95.93	55.57	88.30	53.80	71.62	95.00	No difference	Flavonoid/anthocyanin synthesis pathway
12	*c117196_g1*	*ANS*	336.02	156.6	228.65	63.21	113.07	215.08	No difference	Flavonoid/anthocyanin synthesis pathway
13	*c161528_g2*	*5,3GT*	8.47	7.46	8.88	1.27	1.74	2.45	No difference	Flavonoid/anthocyanin synthesis pathway
14	*c167438_g1*	*3AT*	533.38	169.4	438.8	136.09	198.22	298.18	No difference	Flavonoid/anthocyanin synthesis pathway
15	*c105331_g2*	*URT*	1.15	0.21	0.3	0.12	0.19	0.42	No difference	Flavonoid/anthocyanin synthesis pathway
16	*c160759_g1*	*PSY2*	4.30	4.57	4.67	9.07	8.38	6.56	No difference	Carotenoid synthesis pathway
17	*c172816_g1*	*PDS*	126.86	58.03	110.79	91.24	100.89	84.34	No difference	Carotenoid synthesis pathway
18	*c168460_g1*	*ZDS*	184.16	119.93	212.84	204.53	221.12	211.14	No difference	Carotenoid synthesis pathway
19	*c168442_g1*	*LCYB*	16.36	9	15.37	11.05	16.67	16.53	No difference	Carotenoid synthesis pathway
20	*c144636_g1*	*ZEP*	26.3	15.02	25.09	18.24	25.11	24.57	No difference	Carotenoid synthesis pathway
21	*c143882_g1*	*VDE*	7.53	5.65	8.93	6.97	13.28	10.93	No difference	Carotenoid synthesis pathway

^∗^BHWY represents the sample of the white variant of the Southwest iris (*I. bulleyana* Dykes f. alba YT Zhao), while LHWY represents the Southwest iris (blue).

## Data Availability

RNA-seq data is available at the SRA database in National Center of Biotechnology Information with the accession number PRJNA676187 (https://www.ncbi.nlm.nih.gov/bioproject/?term=PRJNA676187).

## Abbreviations

3GT: Anthocyanin 3-*O*-glucosyltransferase
5GT: Anthocyanin 5-O-glucosyltransferase
CHS: Chalcone synthase
F3H: Flavanone-3-hydroxylase
CHI: Chalcone isomerase
DFR: Dihydroflavonol 4-reductase
ANS: Anthocyanidin synthase
DEGs: Differentially expressed genes
Gb: Gigabites
NT: NCBI nucleotide sequences
NR: NCBI nonredundant proteinsequences
KOG: EuKaryotic Ortholog Groups
GO: Gene Ontology
PFAM: Protein family
qRT-PCR: Quantitative reverse transcription PCR.

## Supplementary Materials

Supplementary Materials Additional File 1: flavonoid targeted metabolomics. (a) Clustering of identified metabolites in Southwest iris (LHYW) and its white variant (BHYW). (b) KEGG enrichment map of different metabolites. (c) KEGG enrichment for flavonoid metabolic pathways. Table S2: top 20 most abundant metabolites in pitaya fruit peel. Table S3: top 20 most abundant metabolites in pitaya fruit pulp. Additional File 2: flavonoid profile of Southwest iris (LHYW) and its white variant (BHYW). Additional File 3: histogram of identified carotenoids and their corresponding levels in Southwest iris (LHYW) and its white variant (BHYW). Additional File 4: targeted metabolomics for carotenoids and identified carotenoids between BHYW (white) and LHYW (Southwest blue iris). Additional File 5: metabolic profile of carotenoids and identified carotenoids between BHYW (white) and LHYW (Southwest blue iris). Additional File 6; summary of sequencing data quality. Additional File 7: list of differentially expressed genes (DEGs) identified as a result of the transcriptomic study of Southwest iris. Additional File 8: list of primers used for qRT-PCR.
