# Supplementary material for: Transcriptome Analysis of Key Genes Involved in Color Variation between Blue and White Flowers of Iris bulleyana
Source: Biomed Res Int. 2023 Jan 18;2023:7407772. doi: 10.1155/2023/7407772 (PMC9876678; doi:10.1155/2023/7407772)
Supplement: Supplementary Materials — Additional File 1: flavonoid targeted metabolomics. (a) Clustering of identified metabolites in Southwest iris (LHYW) and its white variant (BHYW). (b) KEGG enrichment map of different metabolites. (c) KEGG enrichment for flavonoid metabolic pathways. Table S2: top 20 most abundant metabolites in pitaya fruit peel. Table S3: top 20 most abundant metabolites in pitaya fruit pulp. Additional File 2: flavonoid profile of Southwest iris (LHYW) and its white variant (BHYW). Additional File 3: histogram of identified carotenoids and their corresponding levels in Southwest iris (LHYW) and its white variant (BHYW). Additional File 4: targeted metabolomics for carotenoids and identified carotenoids between BHYW (white) and LHYW (Southwest blue iris). Additional File 5: metabolic profile of carotenoids and identified carotenoids between BHYW (white) and LHYW (Southwest blue iris). Additional File 6; summary of sequencing data quality. Additional File 7: list of differentially expressed genes (DEGs) identified as a result of the transcriptomic study of Southwest iris. Additional File 8: list of primers used for qRT-PCR. [file 7407772.f1.zip › Additional file 1.3.pdf]

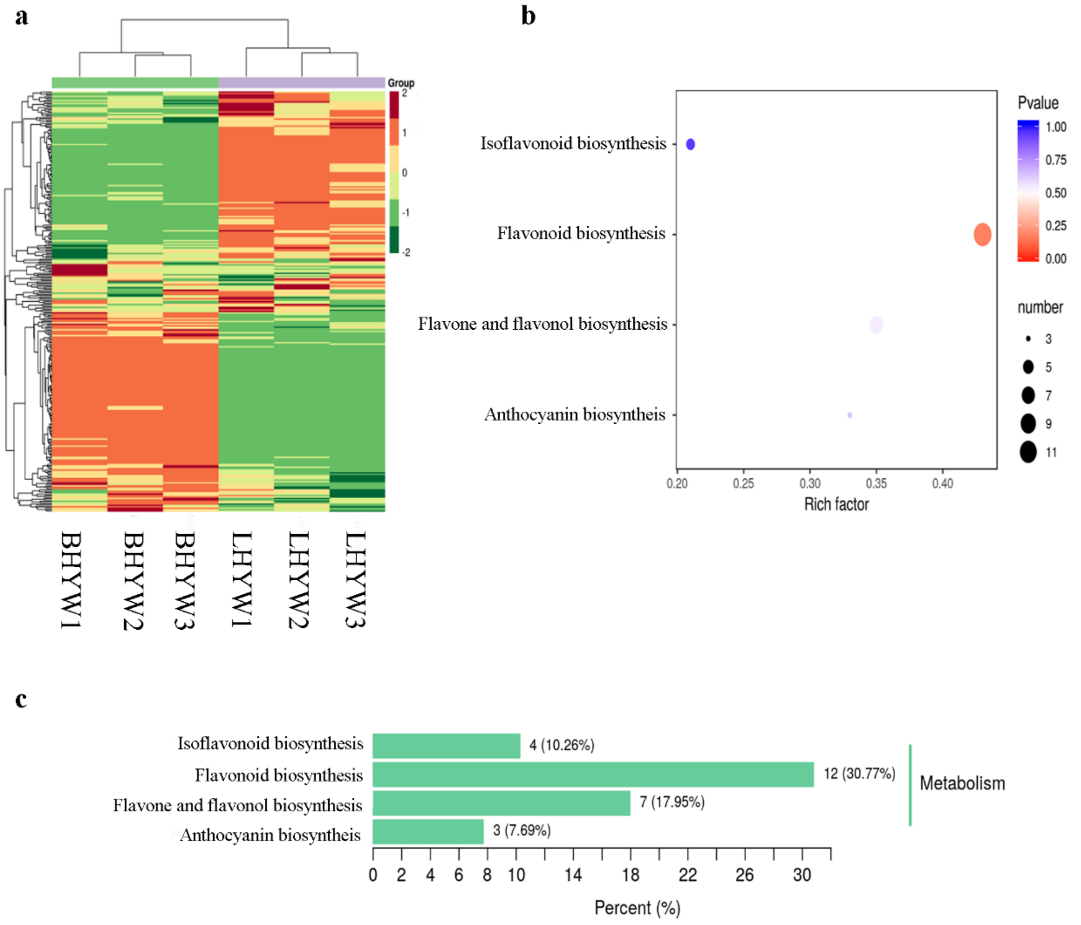

Additional file 1. Flavonoid targeted metabolomics a) clustering of identified metabolites in Southwest iris (LHYW) and its white variant (BHYW) b) KEGG enrichment map of different metabolites c) KEGG enrichment for flavonoid metabolic pathways

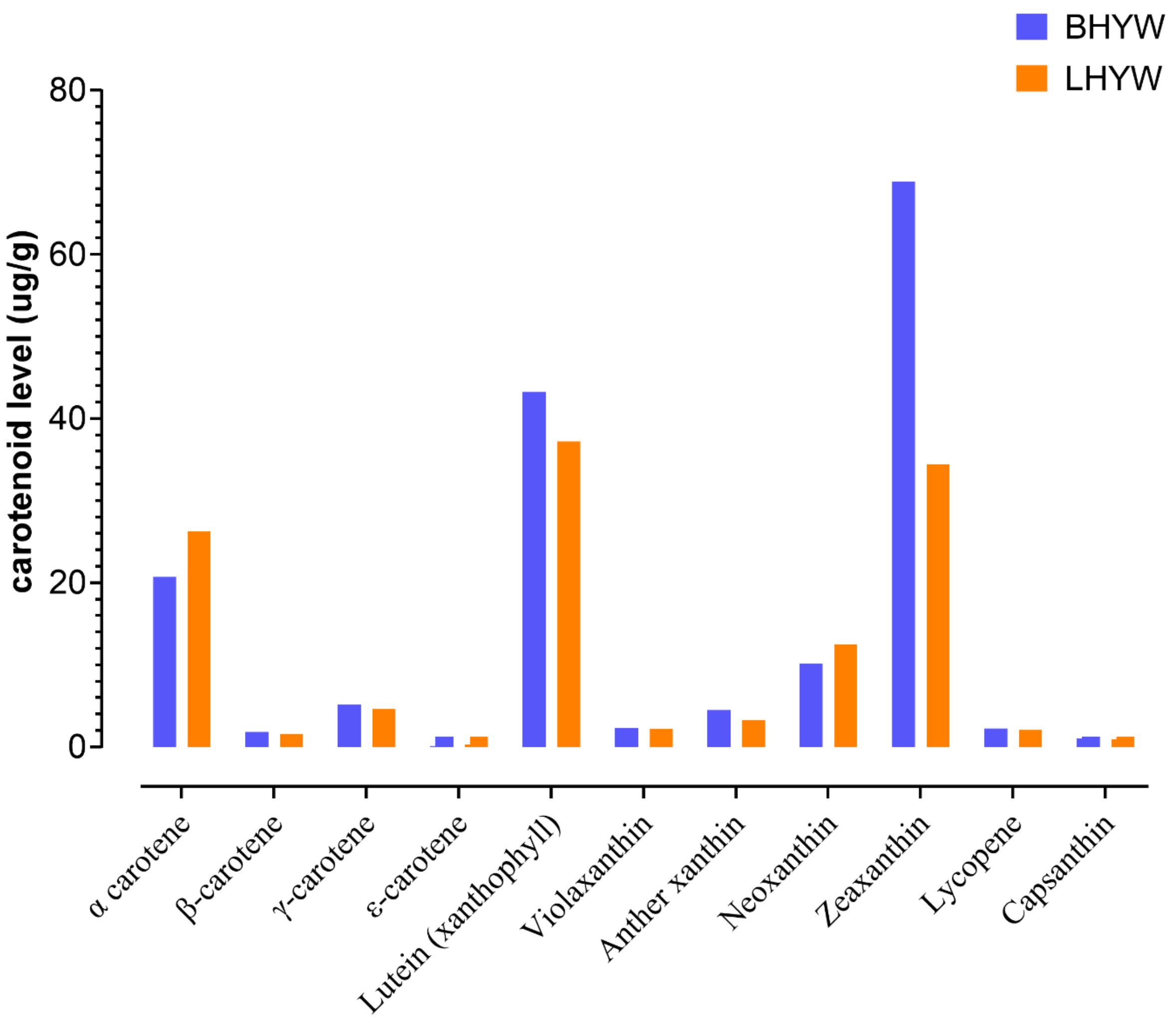

Additional file 3. Histogram of identified carotenoids and their corresponding levels in Southwest iris (LHYW) and its white variant (BHYW);
